# Two Cases of Nasal Rhinosporidiosis in Dogs Treated Endoscopically with a Diode Laser

**DOI:** 10.3390/ani16050737

**Published:** 2026-02-27

**Authors:** Giulia Maggi, Davide De Lorenzi, Enrico Bottero

**Affiliations:** 1 Department of Veterinary Medicine, University of Perugia, Via San Costanzo 4, 06126 Perugia, Italy; 2 San Marco Veterinary Clinic and Laboratory, Via dell’Industria 3, 35030 Veggiano, Italy; 3 Veterinary Clinic “Città di Torino”, Corso Traiano 99, 10135 Torino, Italy

**Keywords:** rhinoscopy, diode laser, minimally invasive surgery, rhinosporidiosis, nasal infection, dogs

## Abstract

Nasal rhinosporidiosis is a chronic granulomatous infectious disease that affects the mucous membranes of the nose and nasopharynx in humans and animals. Although it is endemic in tropical and subtropical regions, sporadic cases have been reported worldwide, including a few cases in Italy. The aim of this report is to describe two cases of nasal rhinosporidiosis in dogs from the same geographical region (Northwestern Italy) that were treated using a diode laser under endoscopic guidance. Both dogs presented persistent respiratory signs, including nasal wheezing, sneezing, and unilateral mucous, catarrhal and hemorrhagic discharge. Both dogs underwent diagnostic procedures, including rhinoscopy, which revealed abnormal tissue growth. Histopathological examination confirmed a diagnosis of proliferative-polypoid rhinitis caused by *Rhinosporidium seeberi* in both cases. In one dog, medical therapy with itraconazole failed to resolve the fungal infection. A local endoscopically guided treatment using a diode laser was thus proposed and successfully performed for polyp removal in both animals. Post-surgical rhinoscopy showed no tissue regrowth in one dog. The second dog experienced recurrence and progression of clinical signs following the first treatment. A second intervention was therefore needed, along with additional oral medical therapy to resolve the fungal rhinopathy. Both dogs were monitored for a minimum follow-up period of one year. Endoscopic debridement is a feasible, minimally invasive technique that could represent a new therapeutic option for the removal of nasal polyps caused by *R. seeberi*.

## Simple Summary

Nasal rhinosporidiosis is a rare infection that affects both people and animals, including dogs, and can cause abnormal tissue growth inside the nose. Although it is more common in warm countries, a few cases have also been reported in Italy. In this case report, we describe a minimally invasive treatment guided by an endoscope in two dogs with clinical signs and diagnostic findings consistent with this nasal infection. In one dog, the treatment completely resolved the disease. In the second dog, the infection returned, requiring an additional procedure to remove the overgrown tissue and medical treatment with antifungal drugs. These results suggest that endoscopic removal of the abnormal tissue using a laser may be a less invasive surgical option for treating this condition.

## 1. Introduction

*Rhinosporidium seeberi* is a Mesomycetozoa (Subkingdom Neozoa, Phylum Neomonada, Class Mesomycetozoea, Order Dermocystida) that causes nasal polyps in dogs [1,2,3]. This organism thrives in humid and aquatic environments such as ponds, rivers, rice fields, and moist soils [4]. The spores released by the sporangia are the infectious form of the organism in the environment. Infection occurs when a person or animal is directly exposed to spores in contaminated water or soil. Upon penetrating the nasal mucosa, and under favorable conditions, the pathogen can lead to the formation of neoformations, which consist of sporangia and reactive granulation tissue [4]. The clinical signs of rhinosporidiosis in dogs include sneezing, nasal wheezing, and unilateral seropurulent or hemorrhagic nasal exudate. These signs are caused by the development of nasal polyps, which may occasionally protrude and become visible outside the nares [3]. Polyps are characterized by an irregular surface, a reddish color, and numerous white–yellow pinpoint foci, which give the mass a typical “strawberry appearance” [3,5]. Nasal rhinosporidiosis is diagnosed through cytological and histopathological examinations; the latter is performed on tissue samples obtained via surgical or endoscopic excision [3,5]. Histological examination of nasal polyps caused by *R. seeberi* shows hyperplasia and erosion/ulceration of the epithelium, along with the growth of fibrovascular tissue containing mixed inflammatory cells [3]. These tissues also contain fungal spores in various stages of development [3], which can be observed also on cytological examination [5]. The treatment of choice for nasal polyps caused by *R. seeberi* is radical surgical excision [5]. Medical therapy with various antifungals (ketoconazole, dapsone, and cefpodoxime proxetil) has been studied, with contrasting results [6]. Surgical intervention can be performed via dorsal rhinotomy with an incision extending through the lateral rhinarium to the nasal septum to include lesions located rostrally [5]. Rhinotomy is an invasive surgical procedure that has been associated with potential intra- and post-operative complications, and requires the hospitalization of the animals [7,8]. This paper describes two cases of canine rhinosporidiosis which occurred in 2024 in Italy (Europe) and were successfully treated with endoscopic removal using diode laser debridement.

## 2. Case Presentation

### 2.1. Case 1

**Figure 1:**
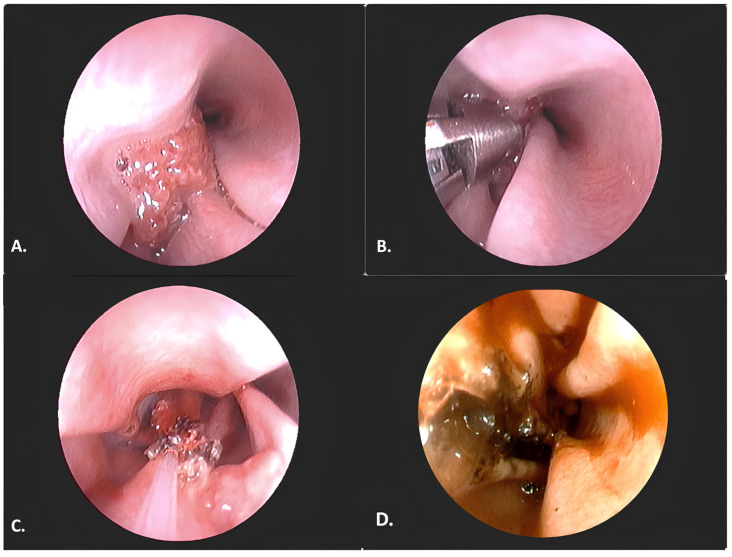
Endoscopic findings of nasal neoformation caused by *Rhinosporidium seeberi* in Case 1 (4-year-old, female Labrador). (**A**) Endoscopic image of a fungal granuloma located in the cranial portion of the middle meatus, dorsal view. The tissue shows a distinctive mottled appearance with whitish areas and a friable consistency. (**B**) Endoscopic view of sampling performed with a coaxial biopsy forceps. (**C**) Endoscopic view of the neoformed tissue during carbonization with a diode laser inserted through the working channel. (**D**) Nasal cavity after endoscopic debridement. The middle meatus is free of the fungal granuloma, with residual necrotic-hemorrhagic material still present.

In May 2024, a 4-year-old female Labrador from the northwest of Italy (Pavia), with both indoor and outdoor access, was presented with a one-month history of recurrent sneezing and unilateral left-sided nasal mucous and catarrhal discharge. One week prior to presentation, the discharge had become tinged with blood. The owner also reported that the dog had been breathing loudly and snoring while asleep. Basic laboratory (complete blood count (CBC) and serum chemistry) examinations were performed, along with serological tests for *Leishmania* spp., which yielded negative results. Due to the suspicion of vegetal foreign body, a rhinoscopic examination was proposed. Inspection of the left nasal cavity with a flexible video-endoscope (EG-250PE5 Video Gastroscope, distal diameter 8.1 mm, working channel 2.2 mm, length 140 cm; Fujifilm, Tokyo, Japan) revealed newly formed tissue, which was irregular in shape and approximately one cm wide, with a reddish color and white dots (Figure 1A).

**Figure 2:**
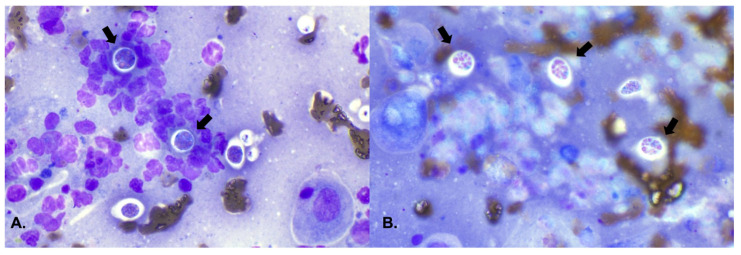
Squash preparation. (**A**) On a moderately proteinaceous background, numerous neutrophils (some showing degenerative features) surround two endospores (10–15 μm) of *Rhinosporidium seeberi* (indicated by an arrow). The endospores show a round to oval morphology with a thin, achromatic capsule. Internally, the nucleus contains multiple characteristic eosinophilic bodies (spherules). (**B**) Three endospores of *Rhinosporidium seeberi*, indicated by an arrow, within a proteinaceous background.

The neoformation was located in the cranial portion of the middle meatus, extending approximately 2 cm caudally. The remaining nasal passages, the right nasal cavity, and the nasopharynx appeared only slightly hyperemic. Multiple samples were collected for cytological and histological examinations. Cytological examination, performed with the squash preparation technique [9] on samples obtained using endoscopic forceps, revealed a weakly hemorrhagic background with a mixed inflammatory cell population, eosinophilic mucoid material, and large, thick-walled spherical structures containing numerous endospores (Figure 2).

**Figure 3:**
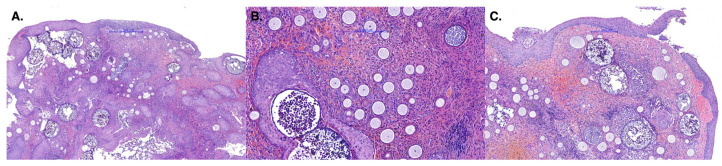
Nasal mucosa (vestibule) showing numerous fungal sporangia at various stages of maturation, which are associated with epithelial hyperplasia, hemorrhage, and a mixed inflammatory process. (**A**) H&E, 500×, scale bar = 500 µm; (**B**,**C**) H&E, 200×, scale bar = 200 µm.

Histological examination revealed proliferative polypoid rhinitis characterized by fibrovascular tissue and intralesional organisms at various stages of maturation, which were consistent with *R. seeberi*. The organisms appeared as spherical structures with an approximately 2 µm-thick eosinophilic wall, containing numerous round spores (5–10 µm in diameter) dispersed within an eosinophilic matrix. (Figure 3).

Medical therapy was initiated with itraconazole at a dosage of 10 mg/kg once daily, pending local treatment, which was performed three weeks after the histological diagnosis, due to the presence of persistent nasal clinical signs. Endoscopic ablation was proposed for the removal of the nasal polyp. The debulking procedure was performed under direct visualization using a flexible video-endoscope (EG-250PE5 Video Gastroscope, distal diameter 8.1 mm, working channel 2.2 mm, length 140 cm; Fujifilm, Tokyo, Japan), alternating between a diode laser (Quanta System^®^, Varese, Italy; fiber diameter 400–600 µm, continuous wave, wavelength 980 nm) (Figure 1B), external grasping forceps (Karl Storz^®^ rigid biopsy forceps, oval, 3 mm, length 150 mm, cod. 69133, Karl-Storz Endoscopy, Tuttlingen, Germany) (Figure 1C), and pressure irrigation, as previously described [10]. No intraoperative or postoperative complications were observed. The dog progressively improved over the following weeks, with resolution of the nasal discharge and disappearance of the sneezing. Post-surgical rhinoscopy, performed 6 weeks after the procedure, showed no evidence of tissue regrowth.

### 2.2. Case 2

In August 2024, a 2-year-old male Golden Retriever from northwest Italy (Vigevano) with both indoor and outdoor access, presented with a one-month history of sneezing, and unilateral left-sided nasal mucous-hemorrhagic discharge. Basic laboratory examinations (CBC and serum chemistry) including serological tests for *Leishmania* spp. and *Filaria* spp. revealed no abnormalities, and the dog subsequently underwent a radiographic and rhinoscopic examination. The radiographic examination, performed in a ventrodorsal open-mouth projection, revealed a slight asymmetry between the nasal cavities and an increase in radiopacity in the cranial portion of the left nasal cavity. The skyline projection for the evaluation of the frontal sinuses appeared normal and symmetrical. Rhinoscopy revealed newly formed highly friable tissue in the left nasal cavity, originating from the mucocutaneous junction and completely occupying the middle and dorsal meatus in the cranial third. The neoformation was irregularly shaped, approximately 3–4 cm wide, with a reddish color, white pinpoint foci, moderate friability during sampling, and bled easily upon contact with the instrument. Multiple biopsies were performed under endoscopic visualization. Cytological and histological examinations, as in Case 1, were diagnostic, and revealed a benign polypoid mass secondary to *R. seeberi* infection. Endoscopic debulking was performed at the time of the first nasal examination, following the results of the cytological analysis. The procedure was carried out using the same approach as for Case 1. No intra-operative or post-operative complications were noted. Post-surgical rhinoscopy was performed 8 weeks after the procedure, and showed no tissue regrowth. In the weeks following treatment, the patient improved clinically. However, after approximately 4 weeks, the dog developed noisy breathing and occasional nasal discharge with intermittent epistaxis. Approximately 14 weeks (≅3.5 months) after the first endoscopy, a second rhinoscopic examination was performed, which revealed recurrence and progression of the initial condition, with newly formed tissue extending to the ethmoturbinates and the ventrocaudal nasal meatus. A second debulking procedure was performed using a diode laser in combination with coaxial forceps to remove the granulomatous fungal tissue. At the end of the procedure, a 1% clotrimazole antifungal cream was applied to the nasal cavity following the same technique as described for the aspergillosis treatment [11]. In addition, oral itraconazole therapy was administered for one month. Endoscopic evaluation at the conclusion of therapy revealed a nasal cavity with a normal endoscopic appearance and no evidence of new tissue formation. A follow-up histological examination demonstrated a mild lymphocytic inflammatory response and no fungal elements.

## 3. Discussion

This case series is the first to describe endoscopic debulking as a treatment for nasal polyps caused by *R. seeberi* in dogs. The endoscopic procedure proved to be safe and effective at removing the polyps, and resulted in complete resolution of the disease and no recurrence in one of the two dogs. The second dog required a second intervention and adjunctive antifungal therapy to resolve the mycotic rhinopathy.

Rhinosporidiosis is an infectious disease endemic to South Asia. *R. seeberi* is an organism whose taxonomic status is controversial and still under debate. Although recent classifications using molecular methods place it within the kingdom Protista (Class Mesomycetozoea), it has been considered similar to certain classes of fungi that thrive in humid environments [1,5,12]. In humans the mode of infection is thought to occur through transepithelial penetration following exposure to an aquatic environment, with local traumatic lesions potentially acting as predisposing factors [13,14,15]. Mere contact with the pathogen does not appear to be sufficient to induce granulomatous infection. Instead, the immunological status of the host plays a decisive role, particularly under conditions of local or systemic immunosuppression [3,13,14,15]. In Italy, cases in both humans and animals have been documented since 1925 and, although still considered sporadic, the disease appears to occur more frequently there than in other parts of Europe [5,16,17]. In dogs, the etiopathogenic mechanism is considered similar, with previous studies hypothesizing the involvement of exposure to humid environments, transmucosal penetration, and traumatic injury [3,5,14]. Notably, a previous case of canine rhinosporidiosis in Italy was reported in Lomellina (Pavia), a region in northwestern Italy characterized by extensive rice fields [5]. Similarly, the two cases described in this report originated from the same area, which suggests that in Italy the disease may be more common than previously thought and that geographical location may be an important epidemiological factor. In addition, although no specific association between breed predisposition and nasal rhinosporidiosis in dogs has been reported in the literature, both affected dogs belonged to retriever breeds (Golden and Labrador Retrievers), which have a strong affinity for water, although neither of the cases was used for hunting purposes. Although direct transmission (both interspecific and zoonotic) through spores contained in nasal discharge has been suspected, a definitive route of transmission has not yet been demonstrated, and many authors agree that water and soil are the medium required for transmission [13,18]. The predominance of large breed dogs is consistent with previous reports [19]. In humans and dogs, the disease more frequently affects young to middle-aged subjects, which supports our findings [6]. Presumably, as in other fungal diseases in dogs, host-related predisposing factors, such as local immune vulnerability at the nasal level, may play a pathophysiological role [20,21]. The dogs included in this case report did not exhibit systemic immune vulnerabilities. However, no information regarding nasal mucosal integrity or local immune status was available.

The clinical signs, macroscopic appearance, radiographic and histological findings of nasal rhinosporidiosis in our series were consistent with findings from other studies [3]. As in our two dogs, the nasal discharge is most commonly mucopurulent, although it may also appear serous (particularly in the early stages, before secondary infection) or hemorrhagic [6,17]. Other frequently reported clinical signs include sneezing, snoring, and stertor [5,6,17,20]. Both dogs reported in these case reports had chronic respiratory symptoms that had continued for one month prior to presentation. Systemic causes of rhinitis and epistaxis, such as Leishmaniasis, were excluded based on serological tests. Other diseases that can present with chronic nasal symptoms (e.g., *Ehrlichiosis*, *Anaplasmosis*, and *Rickettsiosis*) were considered unlikely due to the absence of blood test abnormalities and the presence of unilateral discharge [22]. Rhinoscopy is used to exclude differential diagnoses such as foreign bodies, nasal aspergillosis, odontogenic fistulas, and nasal benign or malignant neoplasms. Although rhinosporidial granulomas may resemble neoplastic lesions endoscopically, their typical cranial localization, reddish coloration with whitish foci (strawberry-like appearance), and marked friability are characteristics of rhinosporidiosis. Radiography was performed in only one of the dogs in this case series and revealed increased radiopacity of the nasal cavity associated with the growth of the mass. Interestingly, in a case series described by Cianatti et al. (1998) [5], no abnormalities were detected on radiographic examination, probably due to the low sensitivity and resolution of radiographs. In a recent case series in dogs, computer tomography (CT) proved effective in identifying nasal masses caused by *R. seeberi*, which were not detected on radiographs in one case and were only suspected in another [19]. For this reason, the recommended clinical approach should include a CT scan, which can provide valuable information on the extent of the mass, its relationship with adjacent structures, and potential osteolytic infiltration, as reported in human cases [23]. Unfortunately, neither of the owners of our two cases consented to a CT scan for economic reasons. Due to the difficulty in culturing *R. seeberi*, diagnosis is generally based on cytological and histopathological examinations. In agreement with previous reports, both methods were diagnostic in our cases, thus supporting the reliability of the squash preparation technique for endoscopic sampling of nasal lesions [5,15].

Various treatment modalities have been reported for nasal rhinosporidiosis in both humans and dogs. Recent reports have described resolution of clinical signs associated with rhinosporidiosis in three dogs following endoscopic biopsy, but unfortunately, no information is available regarding the nature of the tissue collection (excisional or incisional) [19]. However, to the best of our knowledge, no previous study has systematically evaluated endoscopic debulking as a therapeutic surgical option for nasal rhinosporidiosis in dogs. In the present cases, this minimally invasive technique was selected due to the recognized invasiveness and morbidity of traditional surgical procedures.

The most frequently reported complications associated with surgical approaches include bleeding, subcutaneous emphysema, edema, and postoperative infection [24]. Moreover, the invasiveness of surgical treatment necessitates hospitalization of the animals and administration of analgesic therapy to manage pain, thus resulting in a longer recovery time compared to minimally invasive approaches [24]. This information led the owners to decline the surgical option, especially given the systemic health of the patients. The recommended management of rhinosporidiosis in humans is surgical excision with cauterization of the base of the lesion to prevent recurrence. Functional endoscopic sinus (FUS) resection potentially supplemented with a diode laser, is currently the primary management strategy for nasal rhinosporidiosis in humans, especially for small, noninfiltrating lesions [20,25]. The use of the laser was thus deemed an appropriate adjunctive tool for therapeutic management in our cases. The treatment was identical to that used by one of the authors (EB) in a previous study for the removal of benign nasal polyps and nasal carcinomas [10,26]. However, it cannot be ruled out that the dog that experienced a recurrence after the initial treatment might have achieved resolution of the disease with a single surgical intervention.

As previously reported in humans and dogs, lesions commonly recur, and it is difficult to determine whether recurrent lesions are due to relapse or reinfection from the environment [23]. The long interval that often elapses before the recurrence of lesions and symptoms, and which is consistent with an incubation period prior to clinical manifestation, supports the hypothesis of reinfection, particularly in endemic areas.

In contrast, a true relapse may be associated with therapeutic inefficacy, as in our patient, and the incomplete removal of polypoid tissue and parasitic elements, which can occur following surgical or endoscopic debridement [19,20]. It is still unclear whether genetically determined abnormalities in cell-mediated immunity play a role in susceptibility to infection and reinfection [20]. The second local treatment was thus supplemented with an antifungal cream, and systemic therapy was initiated after the endoscopic procedure to enhance the antifungal effect. Adjuvant antimycotic drugs, such as griseofluvin, amphotericin B, trimethoprim-sulphadiazine, sodium stibogluconate, ketoconazole, dapsone and cefpodoxime proxetil have been used, although with variable results in rhinosporidiosis in humans and animals [5,6,20,23]. Topical application of tolnaftate and povidone-iodine has been reported in human and veterinary medicine [13,19].

## 4. Conclusions

These results indicate that endoscopic debridement is a feasible technique. It showed minimal surgery-related morbidity and resolution of clinical signs, with recurrence observed in only one of the two treated animals. Additional prospective studies involving a larger number of animals should be conducted to establish the long-term therapeutic effectiveness of this treatment. Endoscopic laser debulking could be considered a new treatment for nasal rhinosporidiosis in dogs.

## Data Availability

The original contributions presented in the study are included in the article; further inquiries should be addressed to the corresponding authors.

## Abbreviations

The following abbreviations are used in this manuscript:

CT	Computed Tomography
H&E	Hematoxylin and Eosin
FUS	Functional endoscopic sinus surgery

## References

[1] Mendoza L., Taylor J.W., Ajello L. (2002). The class Mesomycetozoea: A heterogeneous group of microorganisms at the animal-fungal boundary. Annu. Rev. Microbiol..

[2] Heutte C., Gargala G., Menotti J., Trecourt A., Muraine M., Gueudry J., Gueit I., Cellier L., Thorel D. (2024). Ocular Rhinosporidium seeberi: A case of conjunctival involvement. J. Fr. Ophtalmol..

[3] Easley J.R., Meuten D.J., Levy M.G., Dykstra M.J., Breitschwerdt E.B., Holzinger E.A., Cattley R.C. (1986). Nasal rhinosporidiosis in the dog. Vet. Pathol..

[4] Toner S., Leguillette R., Israel J., Legge C., Samani A.R.E., Kavanagh M., Goodmanson M. (2024). Long-term follow-up of laryngeal *Rhinosporidium seeberi* diagnosed by PCR and treated with laser ablation and voriconazole nebulization in a retired thoroughbred polo horse. Can. Vet. J..

[5] Caniatti M., Roccabianca P., Scanziani E., Finazzi M., Mortellaro C.M., Romussi S., Mandelli G. (1998). Nasal rhinosporidiosis in dogs: Four cases from Europe and a review of the literature. Vet. Rec..

[6] Borteiro C., Etcheverze J., de León N., Nieto C., Arleo M., Debat C.M., Kolenc F., Ubilla M., Freire J., Dutra F. (2018). Rhinosporidiosis in a dog from Uruguay and review of the literature. Braz. J. Vet. Pathol..

[7] Claeys S., Lefebvre J.B., Schuller S., Hamaide A., Clercx C. (2006). Surgical treatment of canine nasal aspergillosis by rhinotomy combined with enilconazole infusion and oral itraconazole. J. Small Anim. Pract..

[8] Andrews C., Aronson L., Church M., Piegols H. (2024). Dorsal rhinotomy in a dog with a chondro-osseous respiratory epithelial adenomatoid hamartoma: A case report. J. Am. Vet. Med. Assoc..

[9] De Lorenzi D., Bertoncello D., Bottero E. (2008). Squash-preparation cytology from nasopharyngeal masses in the cat: Cytological results and histological correlations in 30 cases. J. Feline Med. Surg..

[10] Bottero E., Mussi E., Raponi F., De Lorenzi D., Ruggiero P. (2021). Diagnosis and outcome of nasal polyposis in 23 dogs treated medically or by endoscopic debridement. Can. Vet. J..

[11] Bottero E. (2022). Endonasal mycosis in dogs. Interventional Endoscopy in Dog and Cat.

[12] Penagos S., Zapata N., Castro J.J., Hidron A., Agudelo C.A. (2021). Rhinosporidiosis in the Americas: A systematic review of native cases. Am. J. Trop. Med. Hyg..

[13] Jain S.N., Ramachandra Rao P.V. (1997). Rhinosporidiosis. Indian J. Otolaryngol. Head Neck Surg..

[14] Almeida F.A., Feitoza L.M., Pinho J.D., de Mello G.C.F., Lages J.S., Silva F.F., Silva R.R., Silva G.E. (2016). Rhinosporidiosis: The largest case series in Brazil. Rev. Soc. Bras. Med. Trop..

[15] Gori S., Scasso A. (1994). Cytologic and differential diagnosis of rhinosporidiosis. Acta Cytol..

[16] Ahmed N.A., Sheik M., Girish R. (2013). Rhinosporidiosis: An epidemiological study. J. Evol. Med. Dent. Sci..

[17] Betts R.H., Gopinath N., Thomas T. (1956). Rhinosporidiosis of the bronchus. Br. J. Surg..

[18] van der Coer G.M., Marres H.A., Wielinga E.W., Wong-Alcala L.S. (1992). Rhinosporidiosis in Europe. J. Laryngol. Otol..

[19] Cridge H., Mamaliger N., Baughman B., Mackin A.J. (2021). Nasal rhinosporidiosis: Clinical presentation, clinical findings, and outcome in dogs. J. Am. Anim. Hosp. Assoc..

[20] Doddawad V.G., Singh R., Shivananda S. (2022). A new technique to resolve nasal rhinosporidiosis: A case report with review of literature. Int. J. Surg. Case Rep..

[21] Sharman M.J., Mansfield C.S. (2012). Sinonasal aspergillosis in dogs: A review. J. Small Anim. Pract..

[22] Mylonakis M.E., Saridomichelakis M.N., Lazaridis V., Leontides L.S., Kostoulas P., Koutinas A.F. (2008). A retrospective study of 61 cases of spontaneous canine epistaxis (1998 to 2001). J. Small Anim. Pract..

[23] Morelli L., Polce M., Piscioli F., Del Nonno F., Covello R., Brenna A., Cione A., Licci S. (2006). Human nasal rhinosporidiosis: An Italian case report. Diagn. Pathol..

[24] Schmiedt C.W. (2018). Nasal planum, nasal cavity, and sinuses. Veterinary Surgery.

[25] Ali G.M., Goravey W., Al Hyassat S.A., Petkar M., Al Maslamani M.A., Hadi H.A. (2020). Recurrent nasopharyngeal rhinosporidiosis: Case report from Qatar and review of the literature. IDCases.

[26] Bottero E., Ferriani R., Pierini A., Mussi E., Ruggiero P., Astorina S., De Lorenzi D., Raponi F. (2025). Palliative endoscopic debulking treatment of canine nasal carcinoma—35 cases (2016 to 2019): A retrospective multicentric study. Can. Vet. J..

